# Engineering *Bacillus subtilis* for production of 3-hydroxypropanoic acid

**DOI:** 10.3389/fbioe.2023.1101232

**Published:** 2023-01-16

**Authors:** Abhroop Garg, Carsten Jers, Hee Jin Hwang, Aida Kalantari, Ildze Ventina, Ivan Mijakovic

**Affiliations:** ^1^ Novo Nordisk Foundation Center for Biosustainability, Technical University of Denmark, Kgs Lyngby, Denmark; ^2^ Systems and Synthetic Biology Division, Department of Biology and Biological Engineering, Chalmers University of Technology, Gothenburg, Sweden; ^3^ Department of Molecular Science and Technology, Ajou University, World cup-ro, Yeongtong-gu, Suwon-si, South Korea

**Keywords:** 3-hydroxypropanoic acid, glycerol, biosynthesis, cell factory, synthetic biology, metabolic engineering

## Abstract

3-Hydroxypropionic acid (3-HP) is a valuable platform chemical that is used as a precursor for several higher value-added chemical products. There is an increased interest in development of cell factories as a means for the synthesis of 3-HP and various other platform chemicals. For more than a decade, concentrated effort has been invested by the scientific community towards developing bio-based approaches for the production of 3-HP using primarily *Escherichia coli* and *Klebsiella pneumoniae* as production hosts. These hosts however might not be optimal for applications in e.g., food industry due primarily to endotoxin production and the pathogenic origin of particularly the *K. pneumoniae*. We have previously demonstrated that the generally recognized as safe organism *Bacillus subtilis* can be engineered to produce 3-HP using glycerol, an abundant by-product of the biodiesel industry, as substrate. For commercial exploitation, there is a need to substantially increase the titer. In the present study, we optimized the bioprocess conditions and further engineered the *B. subtilis* 3-HP production strain. Thereby, using glycerol as substrate, we were able to improve 3-HP production in a 1-L bioreactor to a final titer of 22.9 g/L 3-HP.

## 1 Introduction

3-Hydroxypropionic acid, also called 3-hydroxypropionate (3-HP) is a valuable platform chemical used as a precursor in the chemical industry (Andreeßen and Steinbüchel 2010; Jiang et al., 2021). 3-HP is used as a precursor for the industrial synthesis of several higher value-added chemical products, including acrylic acid, acrylamide, methyl acrylate, acrylonitrile (ACN), ethyl 3-HP, 1,3-propanediol, 3-hydroxypropionaldehyde, propiolactone, and malonic acid, as well as biodegradable materials, especially poly(3-hydroxypropionate) (P3HP) based homopolymers and heteropolymers (Andreeßen and Steinbüchel 2010; Jers et al., 2019; Jiang et al., 2021). These platform chemicals are essential for the synthesis of numerous products, such as solvents, cleaning agents, adhesives, paints and coatings, plastics and plastic packaging, resin-based floor polishes, fibers, absorbent diapers, and disinfectants for sterilizing tissue grafts (Andreeßen and Steinbüchel 2010; Matsakas et al., 2018).

The chemical synthesis of 3-HP in petroleum oil refineries is very costly and generates toxic intermediates with serious environmental effects (Matsakas et al., 2018). There are diminishing global crude oil reserves and increasing environmental deterioration due to notorious chemical pollutants from petroleum and petrochemical industries. These circumstances have necessitated the accelerated growth of biorefinery as an alternative source for the synthesis of various platform chemicals (Li et al., 2016). Biorefinery is the optimized biomass usage for the production of biomaterials, biochemicals, and biofuels for chemical and energy applications with substantial benefits of lower costs and reduced environmental impacts (Hingsamer and Jungmeier 2019). In the production of biodiesel, crude glycerol is the most abundant byproduct. For every 10 tons of biodiesel produced *via* transesterification of vegetable oils or animal fats, 1 ton of crude glycerol is recovered (Andreeßen et al., 2010). This in turn has led to a growing interest in crude glycerol as a renewable substrate for the production of 3-HP and other chemicals (Jiang et al., 2018). The bioconversion of crude glycerol to 3-HP occurs *via* microbial-mediated fermentation processes. To date, a wide repertoire of bacteria with diverse biochemical abilities to naturally produce 3-HP from crude glycerol have been identified (Jers et al., 2019). Genetic engineering has been widely employed for the development of improved 3-HP production strains (Li et al., 2016; Jers et al., 2019). Engineering strategies include screening for better production pathway enzymes, developing expression systems for efficiently producing 3-HP and re-allocation of cellular resources to accumulate 3-HP (Zhao and Tian 2021). With respect to engineering, particularly the Gram-negative bacteria *K. pneumoniae* and *E. coli* have been considered. The highest 3-HP titer, 102.6 g/L, was obtained using *K. pneumoniae* (Zhao et al., 2019). In *E. coli*, the highest titer reported is 76.2 g/L (Kim et al., 2020). However, its pathogenic nature is a deterrent for its use as a cell factory (Zhao and Tian 2021; Zabed et al., 2022). Other hosts, such as *Lactobacillus reuteri*, *Pseudomonas denitrificans*, *Corynebacterium glutamicum* and *B. subtilis* have also been applied for 3-HP production generally with lower resulting titers (Zhou et al., 2013; Ramakrishnan et al., 2015; Chen et al., 2017; Kalantari et al., 2017).

As mentioned, the main body of work has been done using the Gram-negative bacteria *K. pneumoniae* and *E. coli*. Considering that 3-HP can find application in diverse fields including food and pharma sector, these hosts can potentially be problematic. Firstly, due to the production of endotoxin that is observed for most Gram-negative bacteria, and secondly, due to the pathogenic origin of the strains (Li and Tian 2015; Zabed et al., 2022). Consequently, it should be desirably to explore and/or engineer new hosts for 3-HP production. We previously demonstrated a potential of the Gram-positive bacterium *B. subtilis* as a host for heterologous expression of the 3-HP synthetic pathway (Kalantari et al., 2017) and this bacterium is generally recognized as safe (GRAS). The 3-HP biosynthetic pathway from *K. pneumoniae* was successfully introduced in *B. subtilis* to redirect its glycerol metabolism towards 3-HP allowing a titer of 10 g/L in shake flask fermentations (Kalantari et al., 2017). This titer is not high enough for commercial exploitation and a combination of strain and process engineering would be needed to substantially improve titers. Several challenges can be targeted such as preventing buildup of the toxic intermediate 3-hydroxypropanal (3-HPA) as well as byproducts, NAD^+^ co-factor requirement of the aldehyde dehydrogenase, and tolerance to substrate and product (Liang et al., 2022; Zabed et al., 2022). Optimization of the production conditions is key to attain the full potential of production strains. To this end, various fermentation conditions such as growth medium, pH, and dissolved oxygen content have been optimized. For instance, in *K. pneumoniae* and *L. reuteri* optimization of the medium composition led to an increase of 80% and 70% in 3-HP production, respectively (Li et al., 2016; Couvreur et al., 2017). Dissolved oxygen could have different effects on 3-HP production where on one hand aerobic conditions are needed for efficient regeneration of the cofactor NAD^+^ but on the other hand the enzyme glycerol dehydratase is sensitive to oxygen (Jers et al., 2019). Using metabolically engineered *E. coli*, the authors were able to improve the production of 3-HP to 76.2 g/L using fed-batch fermentation by controlling the dissolved oxygen levels at 10% (Kim et al., 2020).

In the present study, the *B. subtilis* strain previously engineered to produce 3-HP from glycerol (Kalantari et al., 2017) was utilized with the aim to improve its 3–HP production capacity. Process conditions for 3-HP production in a bioreactor were optimized and the strain was engineered in order to address NAD^+^ co-factor regeneration, and transport of substrates and product. Collectively, this allowed the production of 23.0 g/L 3-HP using an engineered strain in a 1-L bioreactor.

## 2 Materials and methods

### 2.1 Chemicals

3-HP used as a standard was purchased from TCI Europe N.V. (Zwijndrecht, Belgium). All other chemicals were purchased from Sigma-Aldrich (Steinheim, Germany).

### 2.2 Strains and DNA manipulations

All strains used in this study are listed in Table 1. *E. coli* NM522 was used for plasmid propagation. For marker-free disruptions of the genes *spoIIAC* and *lutP* in *B. subtilis*, the plasmid pMAD carrying a thermosensitive replicon was used (Arnaud et al., 2004). Regions up- and downstream from the target gene were PCR-amplified using relevant primers (Supplementary Figure S1; Table 1) and inserted in the pMAD plasmid. The *spoIIAC* mutation was introduced in the *B. subtilis* strain h-syn-KpDhaB-PuuC-Δglpk-ii to generate the strain PS (production strain). In the PS, the gene *lutP* was disrupted to yield PSΔ*lutP*. The pMAD plasmid was also used to integrate the gene *udhA* from *E. coli* under the control of P43 promoter into the *thrC* gene in the PS. The P43 promoter fragment was amplified from the *B. subtilis* genomic DNA while the terminator fragment was amplified from pBS1C plasmid (Radeck et al., 2013).

**TABLE 1:** Strains used in this study.

Strains	Description	References
h-syn-KpDhaB-PuuC-Δglpk-ii	*Bacillus subtilis* recombinant strain	Kalantari et al. (2017)
PS	h-syn-KpDhaB-PuuC-Δ*glpk*-ii + Δ*spoIIAC*	This study
PS(*udhA*)^ox^	PS + Δ*thrC*::P43-*udhA*	This study
PS(*ndh*)^ox^	PS + P43-*ndh*	This study
PSΔ*lutP*	PS + Δ*lutP*	This study
PSΔ*lutP*(*yvrC*)^ox^	PSΔ*lutP* + P43-*yvrC*	This study
PSΔ*lutP*(*glpF*)^ox^	PSΔ*lutP* + P43-*glpF*	This study
PSΔ*lutP*(*lctP*)^ox^	PSΔ*lutP* + P43-*lctP*	This study
PS4mut	PSΔ*lutP* + P43-*yvrC* + P43-*glpF* + P43-*lctP*	This study

Additionally, the pMAD plasmid was used to replace the native promoter of *ndh* in the PS with the P43 promoter. For replacing the native promoters of the genes *glpF*, *yvrC* and *lctP* with P43 promoter, a CRISPR-Cas9-based method was used (Altenbuchner 2016). The 20 bp spacer sequence oligos were annealed and inserted in the plasmid pJOE8999 between the *Bsa*I restriction sites. The repair template (P43 promoter sequence, and up- and downstream regions) was inserted between the *Sfi*I restriction sites. The pJOE8999 plasmid with the spacer and the repair template was used to transform the PS as described earlier (Yasbin et al., 1975). The resulting strains were analyzed by sequencing to confirm the correct insertion.

### 2.3 Media and growth conditions

*E. coli* and *B. subtilis* were routinely cultured in LB medium (10 g/L tryptone, 5 g/L yeast extract, and 5 g/L NaCl) at 37°C. For experiments to test production of 3-HP, two different media were used; a modified M9 (MM9) medium and a fed-batch fermentation (FBF) medium essentially as reported previously by Wang and co-workers (Wang et al., 2011). The growth medium components of MM9 medium are as follows (in g/L): MM9 seed medium: KH_2_PO_4_ 3.0, Na_2_HPO_4_·2H_2_0 8.5, NaCl 0.5, NH_4_Cl 1.0, MgSO_4_·7H_2_0 0.0246, citric acid·H_2_O 0.0021, MnCl_2_·4H_2_O 0.001, CoCl_2_·6H_2_O 0.0006, NaMoO_4_·2H_2_O 0.0006, ZnCl_2_ 0.0017, CuCl_2_·2H_2_O 0.00043, FeCl_3_·6H_2_O 0.00135, CaCl_2_·2H_2_0 0.00147, yeast extract 1.0, glucose 10.0; MM9 initial medium: KH_2_PO_4_ 6.0, Na_2_HPO_4_·2H_2_0 17.0, NaCl 1.0, NH_4_Cl 2.0, MgSO_4_·7H_2_0 0.0492, citric acid·H_2_O 0.0042, MnCl_2_·4H_2_O 0.002, CoCl_2_·6H_2_O 0.0012, NaMoO_4_·2H_2_O 0.0012, ZnCl_2_ 0.0034, CuCl_2_·2H_2_O 0.00086, FeCl_3_·6H_2_O 0.0027, CaCl_2_·2H_2_0 0.00294, yeast extract 5.0, glucose 10.0, glycerol 10.0; MM9 feed medium is same as MM9 initial medium except: glucose 400.0, glycerol 50.0.

The growth medium components of FBF medium are as follows (in g/L): FBF seed medium: KH_2_PO_4_ 1.0, K_2_HPO_4_ 1.65, NaNO_3_ 10.0, NH_4_NO_3_ 5.0, MgSO_4_·7H_2_0 1.5, MnCl_2_·4H_2_O 0.04, ZnCl_2_ 0.0189, FeCl_2_.4H_2_O 0.047, citric acid 0.019, yeast extract 5.0, glucose 20.0; FBF initial medium: KH_2_PO_4_ 4.0, K_2_HPO_4_ 7.5, NaNO_3_ 10.0, NH_4_NO_3_ 5.0, MgSO_4_·7H_2_0 3.0, MnCl_2_·4H_2_O 0.02, ZnCl_2_ 0.00945, FeCl_2_.4H_2_O 0.031, citric acid 0.019, yeast extract 5.0, glucose 20.0, glycerol 10.0; FBF feed medium: KH_2_PO_4_ 5.0, K_2_HPO_4_ 5.0, NaNO_3_ 10.0, MgSO_4_·7H_2_0 2.0, yeast extract 10.0, glucose 650.0, glycerol 50.0.

For shake flask fermentation experiments, a single colony was inoculated in 10 mL FBF seed medium and incubated with shaking at 37°C overnight. This culture was then used to inoculate 50 mL FBF initial medium supplemented with 10 µM isopropyl β-d-1-thiogalactopyranoside (IPTG) and 15 µM coenzyme B_12_, to obtain a final OD_600_ of 0.05. Shake flask cultures were incubated at 37°C, shaking at 200 rpm for either 30 h or 40 h. For measuring cell density (OD_600_) and the concentration of various metabolites, 1 mL culture broth was taken out at the following time-points (h): 0, 6, 24 and 30 (for 30 h experiments) or 0, 16, 24 and 40 (for 40 h experiments). For the latter, the samples were centrifuged (11,000 g for 3 min) and the supernatant stored at −20°C for the HPLC analysis.

Repeated fed-batch fermentation experiments were carried out at 37°C (unless otherwise stated) in a 1-L Sartorius bioreactor. The pH was maintained at 7.0 by adding 2 M KOH automatically. The stirrer speed was set to be minimum 500 rpm and maximum 1200 rpm, and air flow between 0.15 L/min and 1.5 L/min. These two parameters were controlled automatically in order to maintain the desired dissolved oxygen content. To inoculate the fermentor, a single colony was inoculated in 10 mL seed medium (MM9 or FBF as relevant) and incubated 37°C overnight. The next morning, 30 mL seed medium in a 250 mL shake flask was inoculated with the overnight culture to an OD_600_ of 0.05. The culture was grown until it reached an OD_600_ of approximately 3.0. The seed culture was then used to inoculate 400 mL of initial medium in the 1-L bioreactor to an OD_600_ of 0.1. The initial medium was supplemented with 1 mM IPTG, 20 µM Coenzyme B_12_, and seven drops of Antifoam 204 (Sigma-Aldrich). The Prima BT Bench Top Process Gas Analysis Mass Spectrometer (Thermo Fisher Scientific) was used to monitor the carbon dioxide concentration in the off-gas. Feed medium supplemented with 1 mM IPTG and 20 µM Coenzyme B_12_, was successively added to the bioreactor when glucose was depleted, which was indicated by the decline in carbon dioxide concentration in the off-gas. Glucose depletion in the fermentation broth was also verified using the Glucose MQuant™ (Merck Millipore) glucose strips. A volume of feed medium was added so that the final glucose concentration of was 10 or 20 g/L (as in the corresponding initial medium). Before adding the feed media, 2 mL culture broth was taken out for measuring the cell density and the concentration of select metabolites. Again, the samples were centrifuged (11,000 g for 3 min) and the supernatant stored at −20°C before subsequent HPLC analysis. Experiments were carried out for up to 45 h. In pilot experiments, we did not see any substantial productivity increases after 40 h (data not shown).

### 2.4 HPLC analysis

The concentration of glycerol, 3-HP, glucose, and lactic acid in the growth medium was quantified using HPLC. Samples were centrifuged and filtered through a 0.2 µm sterile filter before the HPLC analysis. 20 µL of sample was run on Aminex HPX-87H ion exclusion column for 45 min. The mobile phase was 0.5 mM H_2_SO_4_ at the flowrate of 0.5 mL/min. The column temperature was set to 65°C, and the refractive index was measured at 35°C. The Shodex RH-101 refractive index detector was used to detect 3-HP, glucose and glycerol. The analytes were identified and quantified by comparing the chromatogram with the respective standards (glycerol, 3-HP, glucose, and lactic acid). The primary rationale for including lactic acid, was its similarity with our target product 3-HP. Based on the standards, we could confirm that the method allowed separating the two compounds. Lactic acid was not detected in any of the samples.

### 2.5 Statistical analysis

Shake flasks experiments were performed in biological replicates. One-way analysis of variance and Tukey’s post-hoc test (*α* = 0.05) was performed to infer statistically significant differences between the tested strains with respect to 3-HP production. For the repeated fed-batch experiments, the experiments were repeated twice and a representative experiment is shown.

## 3 Results and discussion

**FIGURE 1:**
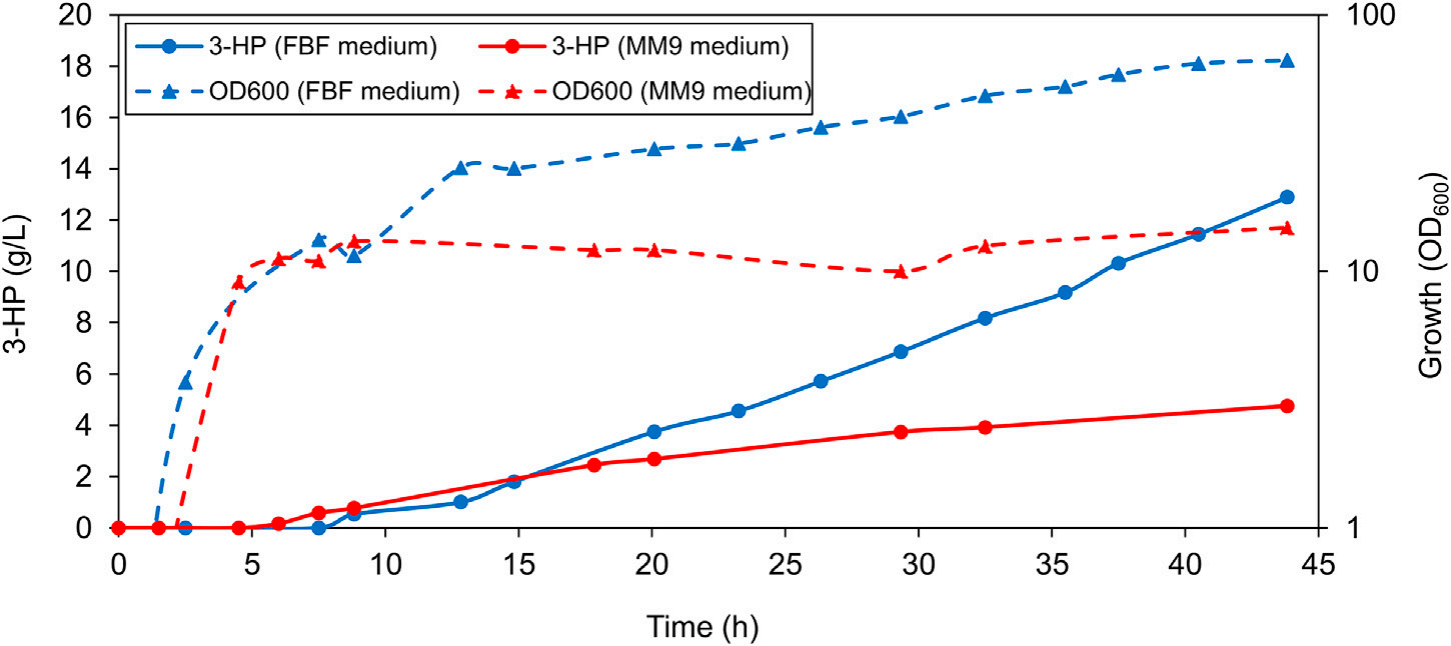
Production of 3-HP in a 1-L bioreactor. Representative graph depicting the concentration of 3-HP and cell density (OD_600_) values of PS grown in fermentation medium compared to the modified 2x M9 medium throughout the 44-h growth period. Cells grown in the fermentation medium exhibited a higher maximum optical density (OD_600_) as well as a higher 3-HP titer.

Before conducting bioreactor experiments, we made a non-sporulating variant of the 3-HP producing strain we previously generated (Kalantari et al., 2017). This was done by inactivating *spoIIAC* which renders *B. subtilis* incapable of sporulation (Kim and Kim 2001). This strain is referred to as the *B. subtilis* production strain (annotated in the text as PS) henceforth. The PS was tested for growth and 3-HP production in a 1-L bioreactor in the MM9 medium. The PS exhibited a maximum cell density with an OD_600_ of 14.8, and 3-HP titer of 4.8 g/L in the MM9 medium (Figure 1). Because of surprisingly low values of cell density and 3-HP production, we then proceeded to test the PS for growth and 3-HP production in the FBF medium that was previously used successfully for riboflavin production in *B. subtilis*. The PS grew better in the FBF medium where it exhibited a higher maximum cell density with an OD_600_ of 66.6, and a higher 3-HP concentration of approximately 12.9 g/L (Figure 1). As the FBF medium enabled both, a higher cell density and 3-HP titer, we selected the FBF medium for all further analyses of the strain.

### 3.1 Optimization of bioconversion process conditions

Having selected a growth medium, we subsequently tested the effect of modifying various fermentation conditions on 3-HP production capacity of the PS in a 1-L bioreactor. For different *B. subtilis* strains, generally optimum temperatures between 30°C and 37°C for growth and/or fermentation activity have been reported (Hoffmann and Bremer 2011; Ju et al., 2019). We thus wanted to probe the temperature-dependence of the process. At 30°C, both the growth (Supplementary Figure S1) and the 3-HP production capacity (4.3 g/L 3-HP) of the PS were significantly reduced as compared to the control (12.9 g/L 3-HP) grown at 37°C (Supplementary Figure S1). This finding indicated that the 3-HP production activity of the PS is temperature-sensitive. The PS needed a favorable temperature of 37°C for optimal growth as previously reported for *B. subtilis* strain 168 (Hoffmann and Bremer 2011).

Next, we tested the effect of IPTG concentration on the 3-HP production capacity of PS. It is well-known that the IPTG concentration can affect the gene expression of IPTG-inducible promoters and consequently target protein concentration. Balancing the expression of the production pathway enzymes could be key in alleviating stresses imposed by e.g., heterologous protein expression and product toxicity due to intracellular accumulation. We tested different concentrations of IPTG (1 μM, 10 μM, 100 μM and 1 mM) in shake flasks for induction of the 3-HP operon, and found that 3-HP production was highest at 10 µM IPTG (Supplementary Figure S2). While the shake flask experiment indicated a potential for improving 3-HP production *via* modulating heterologous gene expression, it did not translate into the same effect in the bioreactor. In the bioreactor setup, the application of 10 µM IPTG led to a reduced production of 3-HP (9.5 g/L 3-HP) (Supplementary Figure S1) as compared to using 1 mM IPTG (12.9 g/L 3-HP).

A potential problem could reside in the stability of production pathway enzymes. The enzyme glycerol dehydratase is prone to being inactivated due to error in regenerating the coenzyme B_12_ cofactor. Glycerol dehydratase reactivase can reactivate the enzyme with help of ATP and intact coenzyme B_12_ (Jers et al., 2019). Thus, the amount of coenzyme B_12_ present can be critical for the continuity of the first step of 3-HP production. We therefore tested the effect of doubling the amount of coenzyme B_12_ on 3-HP production level in a 1-L bioreactor. This led to a slight reduction in 3-HP production (9.2 g/L 3-HP) (Supplementary Figure S1) as compared to the control (12.9 g/L 3-HP) indicating that sufficient coenzyme B_12_ was supplied. Considering that the substrate might be limiting, we also tested if doubling the amount of glycerol (2%) would lead to an improved 3-HP production in the PS. This, however, led to a reduced 3-HP titer (7.4 g/L 3-HP) (Supplementary Figure S1) as compared to the control (12.9 g/L 3-HP).

Finally, we evaluated the 3-HP production capability of the PS under different levels of dissolved oxygen. Oxygen could potentially influence the 3-HP bioprocess by affecting production pathway enzyme stability and co-factor regeneration. A high level of oxygen could lead to inactivation of the oxygen-sensitive enzyme glycerol dehydratase that catalyzes the first step in the 3-HP biosynthetic pathway (glycerol to 3-HPA). On the other hand, the second step of the pathway requires nicotinamide adenine dinucleotide (NAD^+^) as a co-factor, wherein NAD^+^ is reduced to NADH. In an aerobic process, the regeneration of NAD^+^ involves donation of electrons from NADH with oxygen being the final electron acceptor in the electron transport chain. Thus, lowering oxygen level might impact the availability of NAD^+^, thereby affecting the second enzymatic step. Additionally, being a facultative anaerobe, *B. subtilis* generally grows better under aerobic conditions (Hoffmann et al., 1995). Thus, we tested 3-HP production capability of the PS in a 1-L bioreactor under reduced oxygen environment (growth at dissolved oxygen levels of 40%, 20%, 10%, and 1%), but not under anaerobic environment. Surprisingly, no remarkable differences were observed in neither 3-HP production, nor in the growth profile of the cells (Supplementary Figure S3) when grown under different dissolved oxygen conditions. The 3-HP titers were 10.1, 10.4 and 14.2 g/L at dissolved oxygen levels of 20, 10% and 1%, respectively, compared to 12.9 g/L with 40% DO. These findings suggest that either only a minimal amount of oxygen is needed for the regeneration of NAD^+^ from NADH, or other alternative processes are being utilized for regeneration of NAD^+^, especially under near-anaerobic conditions. During anaerobic regeneration of NAD^+^, organic molecules can be used to regenerate NAD^+^ from NADH. For instance, NADH can aid the conversion of pyruvate into lactic acid, and is oxidized to NAD^+^ in the process (Ramos et al., 2000). During alcohol fermentation, alcohol dehydrogenase can oxidize NADH to NAD^+^ while reducing acetaldehyde to ethanol. In the present study, the ability of the PS to regenerate NAD^+^ from NADH under reduced dissolved oxygen growth conditions suggests that oxygen is not critical for this process. Indeed, *B. subtilis* can grow under anaerobic conditions due to their ability to utilize nitrate ammonification and various fermentation processes (Marino et al., 2001). Since the different levels of dissolved oxygen seemed to have almost no effect on the 3-HP production or biomass growth, all subsequent 1-L bioreactor runs were performed under 1% DO.

### 3.2 Genetic modification of *Bacillus subtilis* leads to an improved 3-HP production

Having performed an optimization of the production conditions, we next wanted to assess the possibility of improving 3-HP production *via* genetic engineering of the production strain. Initially we considered the possibility that an increase in NAD^+^/NADH ratio would improve 3-HP production. Consequently, we used different approaches to modulate this ratio. The native NADH dehydrogenase encoded by *ndh*, which can oxidize NADH to NAD^+^ was over-expressed (Marreiros et al., 2016). This was done by placing it under the control of P43 promoter. Additionally, we introduced the enzyme pyridine nucleotide transhydrogenase (UdhA) from *E. coli*, which increases the transfer of reducing equivalents between NAD^+^ and NADP^+^ (Boonstra et al., 1999). Unfortunately, none of these introduced gene components substantially increased 3-HP production in the PS (Supplementary Figure S4). These experiments, in addition to fermentation experiments with different oxygen levels, indicated that the balance of NAD^+^/NADH was not a bottleneck in the 3-HP production in our experiments. Other studies have reported various means, including aearation control, electro-fermentation and enzymatic approaches, to control the redox balance leading to improved 3-HP production (Li et al., 2013; Kim et al., 2017; Kim et al., 2020). While NAD^+^/NADH in our current strain did not appear to be a bottleneck, further improvement in 3-HP production and derived NAD^+^-drain could make it a relevant parameter in a later stage.

**FIGURE 2:**
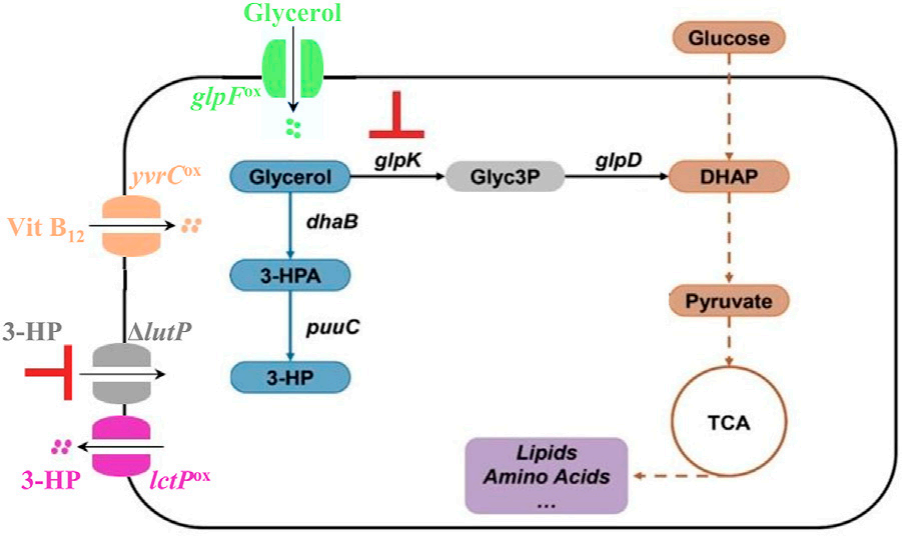
Overview of the genetic modifications performed in the PS. We over-expressed the genes glpF (for improved import of glycerol), *yvrC* (for improved import of coenzyme B_12_), *lctP* (for improved export of 3-HP), and knocked out the gene *lutP* (to import of 3-HP).

As described above, increasing the amount of coenzyme B_12_ and glycerol also did not lead to an increase in 3-HP production. We hypothesized that this could be because of limitations in their uptake by the bacterial cell. Therefore, we made a series of genetic modifications in the PS targeting transporters to increase substrate import (GlpF), product export (LctP), and import of the glycerol dehydratase coenzyme B_12_ (YvrC) as well as preventing import of the product molecule 3-HP (LutP). The genetic modifications performed in the PS are depicted in the Figure 2. *B. subtilis* does not have dedicated 3-HP transporters but due to the structural similarity we hypothesized that the native lactate transporters facilitated the transport of 3-HP. To assure that 3-HP is efficiently secreted, we over-expressed the permease LctP that is responsible for the export of lactate (Ramos et al., 2000; Chai et al., 2009), and hence potentially of 3-HP as well. As 3-HP concentration increases in the fermentation broth, re-uptake of 3-HP could potentially be decreasing the productivity, and with the same rationale, we therefore targeted the main permease for lactate uptake LutP (Chai et al., 2009). To address these transport processes, we inactivated the *lutP* gene to yield the strain PSΔ*lutP*. In this strain, we further over-expressed *lctP* by placing it under the control of the strong promoter P43, thereby obtaining the strain PSΔ*lutP*(*lctP*)^ox^. The influx of the substrate glycerol could also be a potential bottleneck. We therefore overexpressed the glycerol transporter GlpF (Stroud et al., 2003) by placing *glpF* under control of the P43 promoter to yield the strain PSΔ*lutP*(*glpF*)^ox^. Our 3-HP synthesis pathway is dependent on the cofactor coenzyme B_12_. However, in presence of coenzyme B_12_, the uptake is negatively regulated *via* a riboswitch regulating expression of *yvrC* encoding an ABC transporter protein (Nahvi et al., 2004). To improve coenzyme B_12_ uptake, we therefore placed *yvrC* under control of the P43 promoter to obtain PSΔ*lutP*(*yvrC*)^ox^. Finally, we also constructed a strain in which all the four mutations were introduced simultaneously. This strain, PSΔ*lutP*(*yvrC*)^ox^(*glpF*)^ox^(*lctP*)^ox^, is referred to as PS4mut in this manuscript.

**FIGURE 3:**
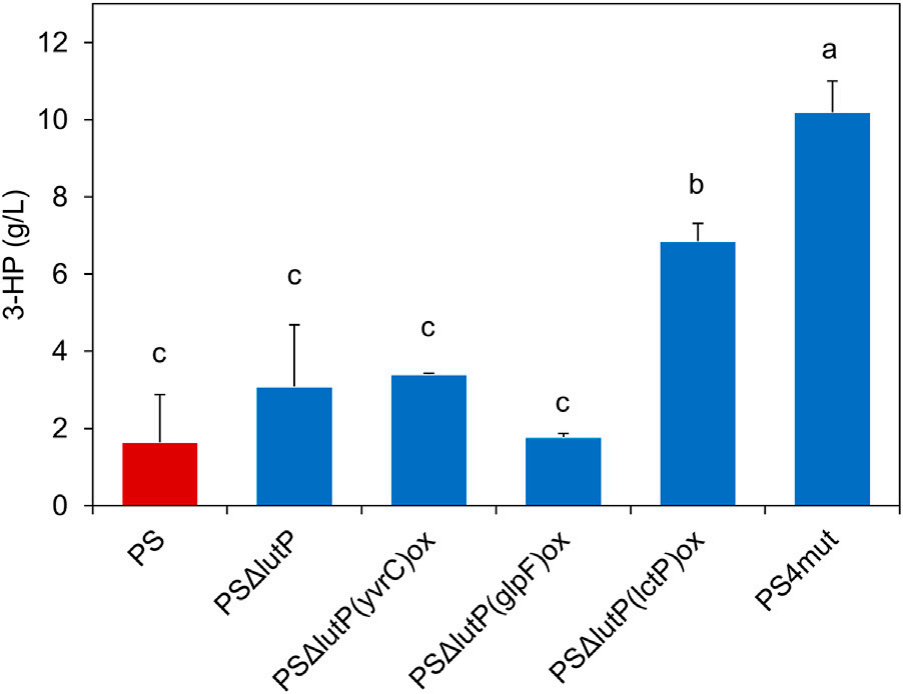
3-HP production in the genetically modified strains PSΔ*lutP*, PSΔ*lutP*(*lctP*)^ox^, PSΔ*lutP*(*glpF*)^ox^, PSΔ*lutP*(*yvrC*)^ox^ and PS4mut compared to the PS grown in shake flasks after 30 h. The “*” represents statistical significance with *p* < 0.05. The error bars represent standard deviation (n = 2). Different roman letters show significantly different means as determined by Tukey’s post-hoc test.

These five engineered strains, i.e., PSΔ*lutP*, PSΔ*lutP*(*glpF*)^ox^, PSΔ*lutP*(*yvrC*)^ox^, PSΔ*lutP*(*lctP*)^ox^ and PS4mut, along with PS (as a reference) were evaluated in shake flasks to determine the effect of these genetic modifications on 3-HP production (Figure 3). The PS produced about 1.6 ± 1.2 g/L 3-HP while the single mutant strain PSΔ*lutP* produced 3.1 ± 1.6. Neither of the double mutants strains PSΔ*lutP*(*glpF*)^ox^, or PSΔ*lutP*(*yvrC*)^ox^ exhibited a significant increase in the 3-HP titer compared to the PS. In the strain PSΔ*lutP*(*lctP*)^ox^ where the aim is to improve export and decrease re-import of 3-HP we observed a significant increase in 3-HP titer (6.9 ± 0.5 g/L). We previously observed no apparent growth defect of *B. subtilis* in presence of ∼9 g/L 3-HP (Kalantari et al., 2017), which could indicate that the primary effect is exerted *via* the improved secretion of 3-HP. Similar benefits, albeit in connection with higher overall 3-HP titers, has been observed in *E. coli* where upregulation of transporter YohJK leads to improved 3-HP tolerance and increased 3-HP production (Nguyen-Vo et al., 2019; Nguyen-Vo et al., 2020). Combining all four mutations led to further significant improvement in 3-HP titer (10.2 ± 0.8 g/L) as compared to the PSΔ*lutP*(*lctP*)^ox^. It is worth noting that the genetic modifications led to altered growth behavior for all strains. For all five mutant strains, we observed a reduced final cell density in the shake flask cultures compared to the PS strain (OD_600_ of 11.2–13.2 vs. 17.7, respectively) (Supplementary Figure S5).

**FIGURE 4:**
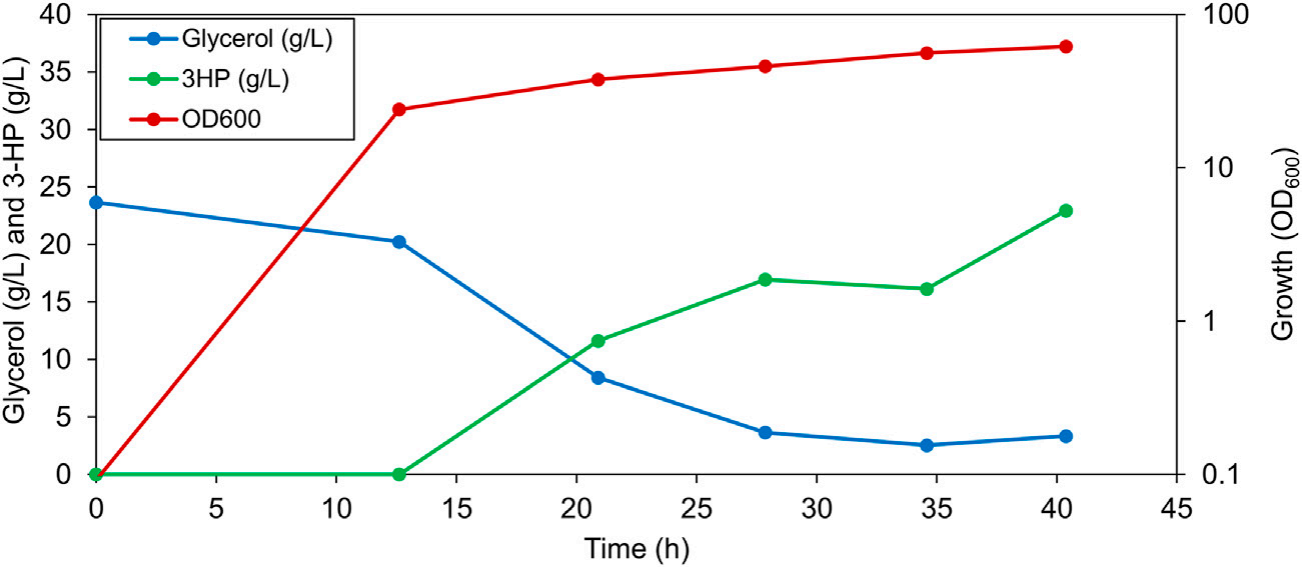
Representative graph depicting 3-HP production, residual glycerol in the broth and the OD_600_ value of PS4mut in a 1-L bioreactor cultured under 1% dissolved oxygen and 2% glycerol.

The shake flask experiments, indicated a potential of the PS4mut strain for the production of 3-HP. Next, we wanted to test the performance of this strain in our 1-L bioreactor setup. Anticipating a higher 3-HP production in the quadruple mutant as compared to the PS, we doubled the amount of input glycerol (2% glycerol) in order for it to not be a limiting factor. As expected, an improved 3-HP production with a titer of about 22.9 g/L was observed in this strain (Figure 4), thus highlighting the importance of optimizing the transport processes in the production strain. By a combination of metabolic engineering of *B. subtilis* and optimization of the bioprocess conditions, we were able to increase 3-HP production by 4.8-fold (from 4.8 g/L in the PS to 22.9 g/L in PS4mut) in a 1-L bioreactor.

## 4 Conclusion and perspectives

The potential of *B. subtilis* as a cell factory has been established earlier (Kalantari et al., 2017). By optimization of the bioprocess conditions and genetic engineering, we have further improved the 3-HP production capacity of *B. subtilis* by 4.8-fold to a maximum titer of 22.9 g/L. This titer is still not high enough for commercial exploitation. Further improvements in 3-HP production could potentially be achieved by improving the resistance of the production strain towards 3-HP and glycerol by adaptive laboratory evolution (ALE). For instance, ALE was used to develop a 3-HP tolerant *E. coli* strain which produced more 3-HP as compared to the reference strain (Nguyen-Vo et al., 2019). In another study, the glycerol assimilating capability of *Cupriavidus necator* was improved by ALE (González-Villanueva et al., 2019). Employment of highly active and specific glycerol dehydratases and alcohol dehydrogenases could be a venue for further improving the 3-HP production. Importance need also be given to the optimization of the bioprocess conditions. Such a combinatorial approach would be needed in order to reach industrially relevant 3-HP titer, yield and productivity.

## Data Availability

The original contributions presented in the study are included in the article/Supplementary Material, further inquiries can be directed to the corresponding author.
