# Supplementary material for: Engineering Bacillus subtilis for production of 3-hydroxypropanoic acid
Source: Front Bioeng Biotechnol. 2023 Jan 16;11:1101232. doi: 10.3389/fbioe.2023.1101232 (PMC9885095; doi:10.3389/fbioe.2023.1101232)
Supplement: Supplementary file 1 [file DataSheet1.PDF]

# Supplementary Material

## 1 Supplementary Figures and Tables

### 1.1 Supplementary Figures

A

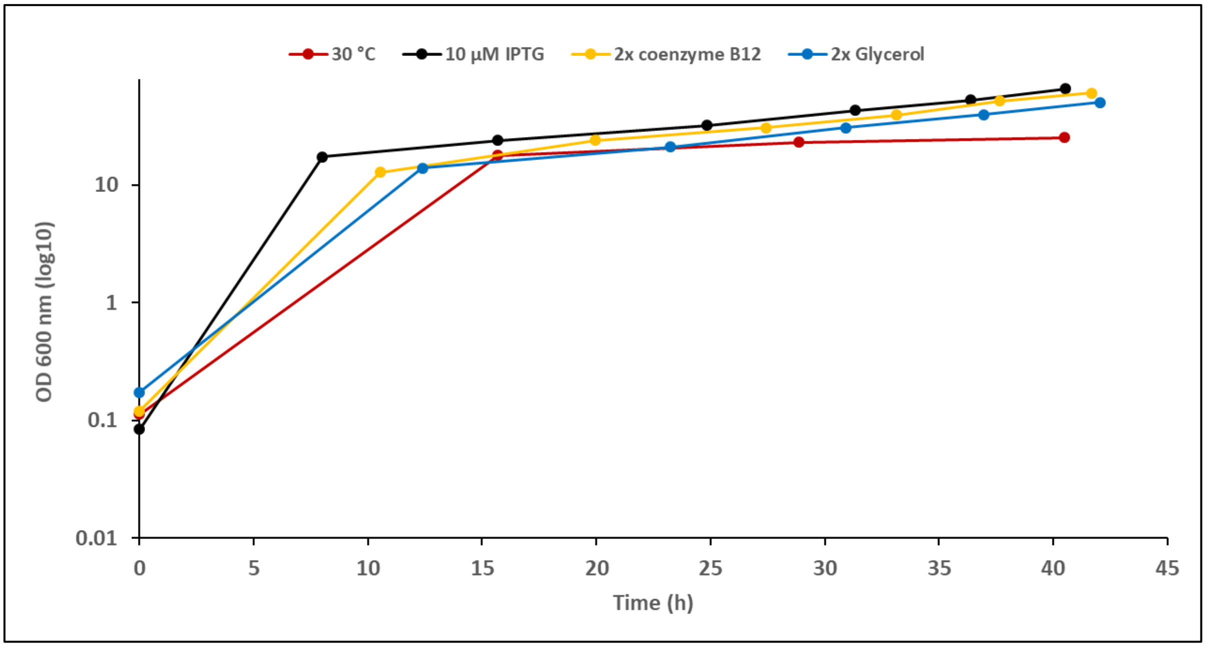


B

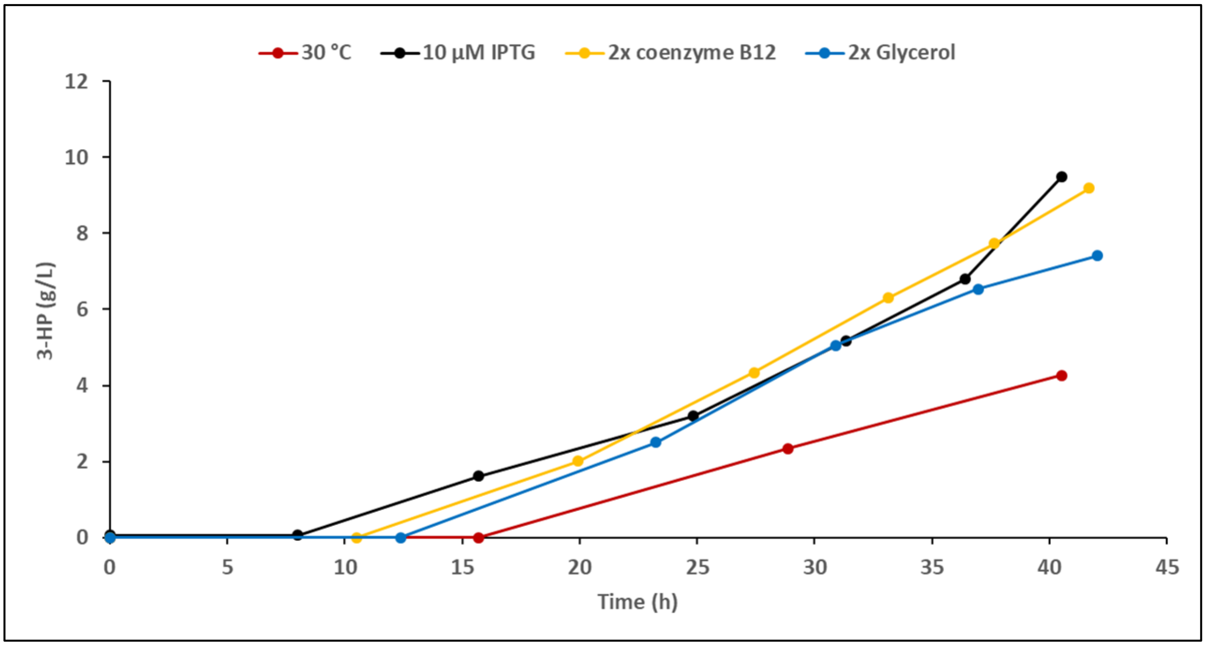


**Supplementary figure 1.** Cell optical density ($OD_{600}$) values (panel A) and 3-HP production (panel B) of the PS cultured under 40 % dissolved oxygen content in a 1-L bioreactor (a) at 30 °C, (b) induced with 10 µM IPTG, (c) with 40 µM coenzyme $B_{12}$, and (d) 2 % glycerol.

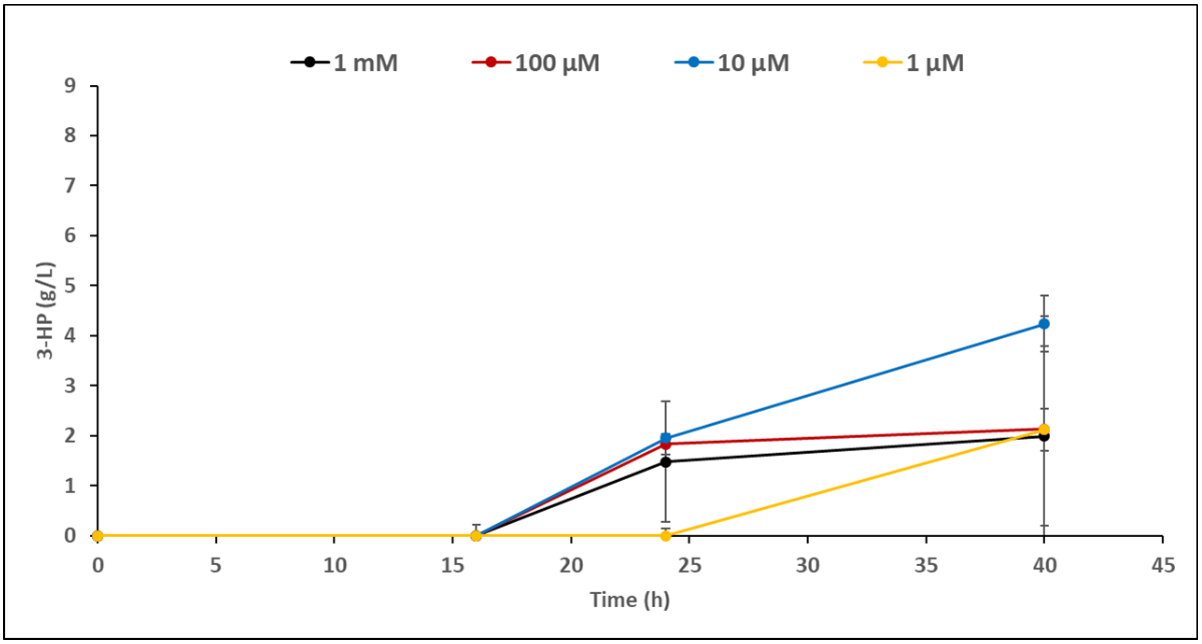


**Supplementary figure 2:** 3-HP production in the PS induced with different concentrations of IPTG cultured in shake flasks.

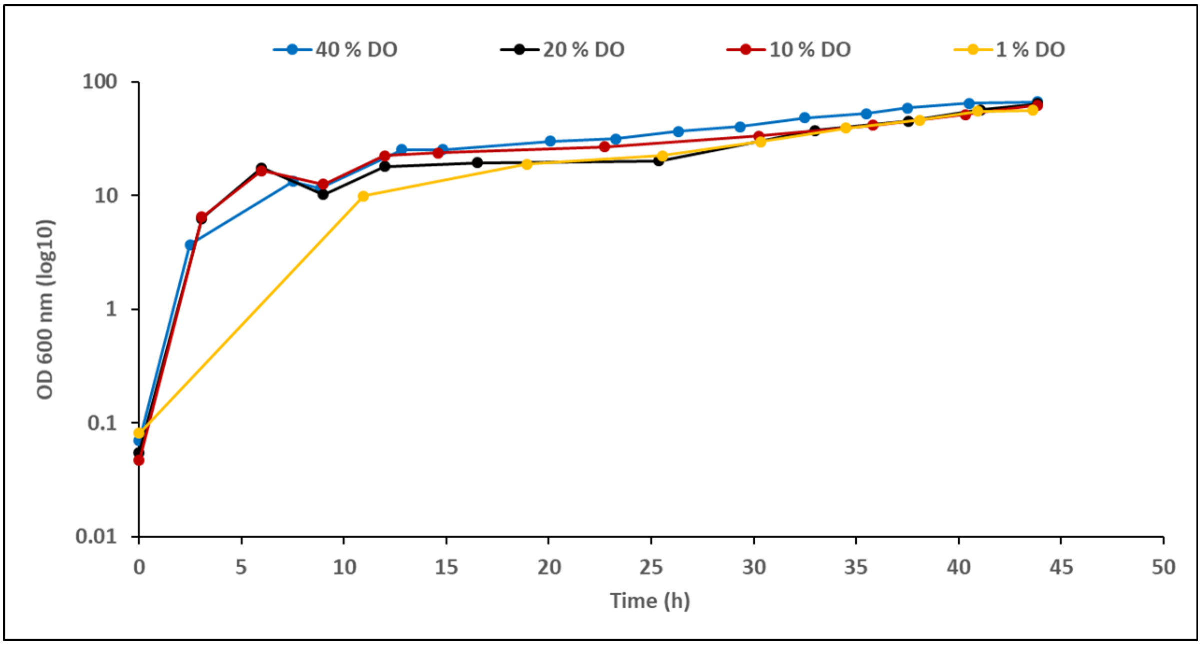


**Supplementary figure 3**. Cell optical density ($OD_{600}$) values of the PS cultured under different dissolved oxygen (DO) content in a 1-L bioreactor, measured at 600 nm.

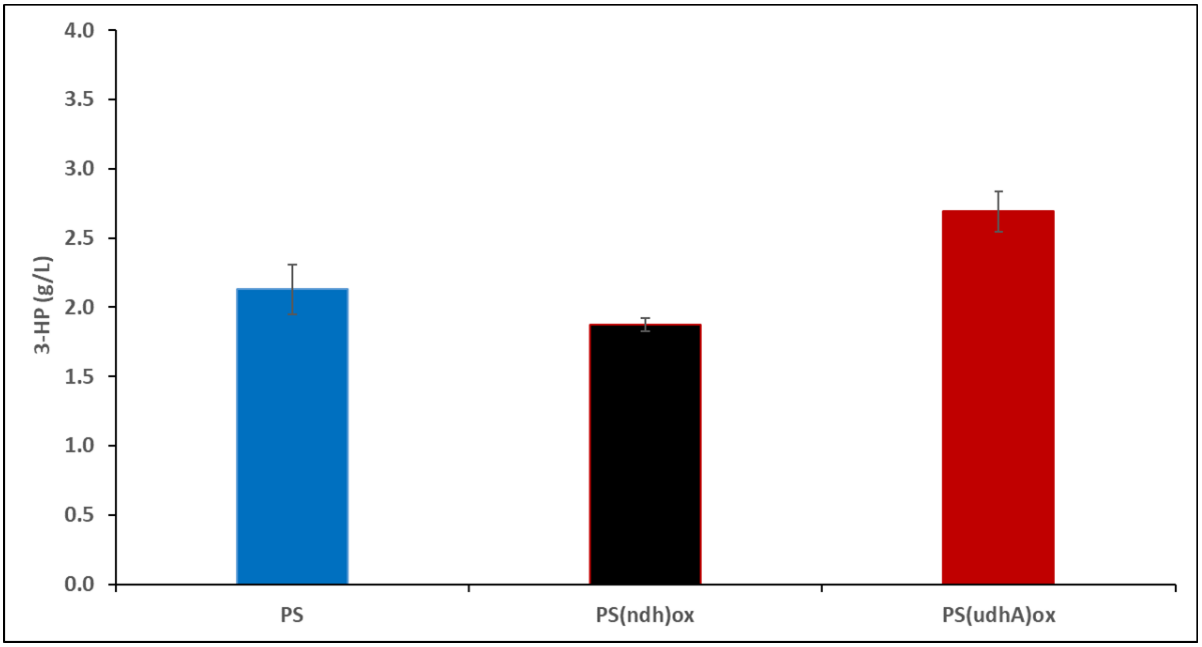


**Supplementary figure 4.** 3-HP production in different strains cultured in shake flasks after 72 h, induced with 1 mM IPTG. The error bars represent standard deviation (n=3).

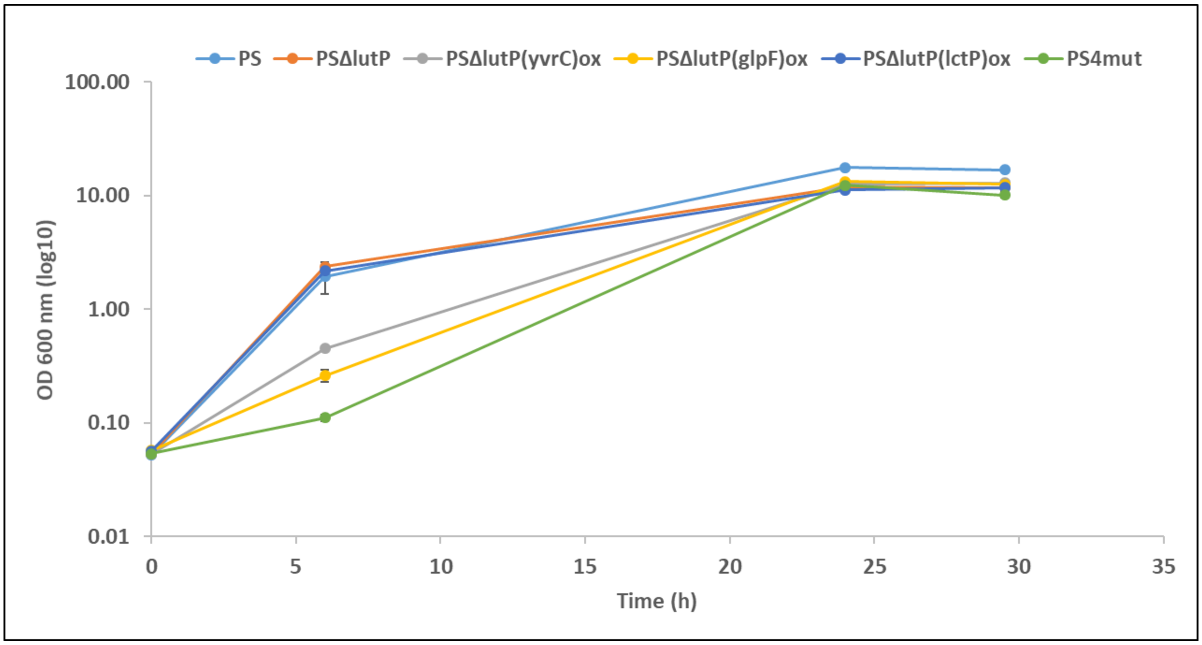


**Supplementary figure 5.** Cell optical density ($OD_{600}$) values of genetically modified strains PSΔ*lutP*, PSΔ*lutP*(*lctP*)ox, PSΔ*lutP*(*glpF*)ox, PSΔ*lutP*(*yvrC*)ox and PS4mut compared to the PS cultured in shake flasks. Dots represent mean $OD_{600}$ values, the error bars represent standard deviation (n=2).

## 1.2 Supplementary Table

**Table S1:** List of primers used in this study.

| Primer | Primer sequence (5’ to 3’) | Remark |
|---|---|---|
| ΔspoIIAC-up-BamHI-F | cgcggatccgaatcgttcgcccgtgtgacagttgc | For generating the strain PS |
| ΔspoIIAC-up-R | gagttgtcagcgat gcagccgatctggaagagatc | |
| ΔspoIIAC-down-F | ccagatcggctgcatcg ctgacaactcagaagaaaaatgg | |
| ΔspoIIAC-down-EcoRI-R | ccggaattccgttgataaatttcgctttcg | |
| ΔspoIIAC-confirm-F | atggatgtggaggttaag | |
| ΔspoIIAC-confirm-R | ctagccatccgtatgatccatttg | |
| Δlutp-up-F | gtctgcagaagcttctattctccctgtttcatttcgac | For generating the strain PSΔlutP |
| ΔlutP-up-R | ccaccgctatcgactaactgtcccgttttcacag | |
| ΔlutP-down-F | agtcgatagcggtggcg | |
| ΔlutP-down-R | cgggagctcgaattcatccggctcctgtcagg | |
| P43-F | gcggcttccttgtagag | For amplifying the P43 promoter fragment |
| P43-R | agtagttcctccttatgtgtgtacattcctctcttac | |
| pBS terminator-F | taacgctgatagtgctagtg | For amplifying the terminator fragment |
| pBS terminator-R | gcggccgctactagtata | |
| ΔthrC-up-F | gtctgcagaagcttctaattcatgtaaaagatgaggttgg | |

| | | |
|---|---|---|
| ΔthrC-up-R | ctacaaggaagccgccgaaggcagcagttttttgg | For generating the strain PS(udhA)$^{ox}$ |
| udhA-F | cacataaggaggaactactatgccacattcctacgatta | |
| udhA-R | ctagcactatcagcgttacatttaaaacaggcggtttaaacc | |
| ΔthrC-down-F | ctagtagcggccgcaaaatgcgcggctttgaagc | |
| ΔthrC-down-R | cgggagctcgaattctgtcgctccacaccttcac | |
| ndh-up-F | gtctgcagaagcttctatttgtgtttgctattttcagag | For generating the strain PS(ndh)$^{ox}$ |
| ndh-up-R | ctacaaggaagccgccgtatatcctccgtccttt | |
| ndh-down-F | cacataaggaggaactactatgtcaaaacatattgtcattc | |
| ndh-down-R | cgggagctcgaattctacatttgtaacaggaagac | |
| P43-RBS-F2 | gtttgtcctccttattagttaatcacctataatggtaccgctatcac | Used as forward or reverse primer for F2 fragments below |
| pJOE-glpF-spacer-F | tacgaatgtgctatgacagcattt | For generating the strain PSΔlutP(glpF)$^{ox}$ |
| pJOE-glpF-spacer-R | aaacaaatgctgtcatagcacatt | |
| pJOE-glpF-F1- SfiI-F | aaggccaacgaggcccccgctgggattatttccac | |
| glpF-F1-R | ctttagcaagaagcacattcctcctaaagtcac | |
| glpF-F2-F | gaggaatgtgcttcttgctaaagcggccaagg | |
| glpF-F3-F | ggtgattaactaataaggaggacaaacatgacagcattttggggagaag | |
| pJOE-glpF-F3-SfiI-R | aaggccttattggccgacgattaaaggattaagtc | |
| pJOE-yvrC-spacer-F | tacgaagattatgaaaaaacgagc | For generating the strain PSΔlutP(yvrC)$^{ox}$ |
| pJOE-yvrC-spacer-R | aaacgctcgttttttcataatctt | |

| | | |
|---|---|---|
| pJOE-yvrC-F1- SfiI-F | aaggccaacgaggcctttttctccgccccgccag | |
| yvrC-F1-R | ggtgattaactaataaggaggacaaacatgaaaaaacgagccgggatatg | |
| yvrC-F2-R | ggaaaagatttcttgctaaagcggccaagg | |
| yvrC-F3-F | ctttagcaagaaatcttttcctcctaaacgaactaaaaaag | |
| pJOE-yvrC-F3-SfiI-R | aaggccttattggccatgactgccgcaccctcttg | |
| pJOE-lctP-spacer-F | tacgaaagggctgattgtcaatgt | For generating the strain PSΔlutP(lctP)$^{ox}$ |
| pJOE-lctP-spacer-R | aaacacattgacaatcagcccttt | |
| pJOE-lctP-F1-SfiI-F | aaggccaacgaggcccagcgcctcaaaacgtacac | |
| lctP-F1-R | cgctttagcaagatgacaatcagccctttactctaaag | |
| lctP-F2-F | ctgattgtcatcttgctaaagcggccaagg | |
| lctP-F3-F | ggtgattaactaataaggaggacaaacatgtgggagcagttgtatgatc | |
| pJOE-lctP-F3-SfiI-R | aaggccttattggccggctccggaggcagcgtttg | |
